# Modelling Speaker Attribution in Narrative Texts With Biased and Bias-Adjustable Neural Networks

**DOI:** 10.3389/frai.2021.725321

**Published:** 2022-02-03

**Authors:** Tillmann Dönicke, Hanna Varachkina, Anna Mareike Weimer, Luisa Gödeke, Florian Barth, Benjamin Gittel, Anke Holler, Caroline Sporleder

**Affiliations:** ^1^Göttingen Centre for Digital Humanities, University of Göttingen, Göttingen, Germany; ^2^Department of German Philology, University of Göttingen, Göttingen, Germany

**Keywords:** narrative understanding, annotation, bias, questionnaire, subjectivity, text classification

## Abstract

Literary narratives regularly contain passages that different readers attribute to different speakers: a character, the narrator, or the author. Since literary narratives are highly ambiguous constructs, it is often impossible to decide between diverging attributions of a specific passage by hermeneutic means. Instead, we hypothesise that attribution decisions are often influenced by annotator bias, in particular an annotator's literary preferences and beliefs. We present first results on the correlation between the literary attitudes of an annotator and their attribution choices. In a second set of experiments, we present a neural classifier that is capable of imitating individual annotators as well as a common-sense annotator, and reaches accuracies of up to 88% (which improves the majority baseline by 23%).

## 1. Introduction

Humans have different habits when it comes to reading and interpreting literature. Therefore, human annotators bring different assumptions and beliefs to the annotation task and introduce an *annotator bias* (e.g., Geva et al., 2019) in the data creation. Annotator bias has been studied in regard to several phenomena of natural language understanding (see Geva et al., 2019; Akhtar et al., 2020; Kuwatly et al., 2020), but not yet in the literary domain. This is surprising, because a basic problem in literary studies is the inability to hermeneutically examine the influence of the recipients' world knowledge on the reception process. Since literary narratives are highly ambiguous constructs, it is often impossible to choose between diverging interpretations of a specific passage using the hermeneutic approach, which is also reflected in the annotation of literary phenomena. Following Soberón et al. (2013), we view annotation disagreements as a valuable source of information that allows us to explore readers' perceptions of texts and the factors influencing these. Concretely, we will show that disagreements regarding the annotation of literary phenomena are not randomly distributed but fall into discernible patterns that can be attributed to differing literature-specific preferences and implicit beliefs of readers. To this end, we compute correlations between questionnaire-based data on readers' preferences and beliefs, and the annotations produced by them. In a second step, we develop a bias-adjusted (and bias-adjustable) classifier, that takes into account literature-specific attitudes of annotators and show that this outperforms an annotator-agnostic classifier.

Section Theoretical Background introduces the concrete literary phenomenon we are dealing with: the attribution of so-called reflective passages to a character, the narrator, or the author of a narrative text of fiction. As the concept of a “reflective passage” is not yet well formalised in literary theory, we annotated three phenomena which we consider to be strong indicators for reflective passages: comment (Bonheim, 1975), non-fictional speech (Konrad, 2017) and generalisation (Leslie and Lerner, 2016). Annotators had to identify these and attribute them to one or more of the three attribution classes (character/narrator/author). In the third section, we introduce our corpus (German narrative fiction from 1650 to 1950) as well as our questionnaire on literary attitudes (which expands the Literary Response Questionnaire from Miall and Kuiken, 1995), we describe procedures of data preparation, feature selection and specify our neural classifier including the different conditions it has been trained under. In the fourth section, we report our results concerning a) correlations between literary attitudes of the annotators and the annotations they produce and b) the classification of attributions by our models. In the last section, we give a short overview over existing work that relates to our study, discuss our results and point out further routes for research.

## 2. Theoretical Background

### 2.1. Reflective Passages

Narrative texts consist of sentences and passages that serve different functions in discourse. Some sentences convey plot elements, describe scenes, or consist of direct character speech. There are also passages that are characterised in particular by the fact that no action is reproduced in them, but rather the impression of a narrative pause (i.e., a pause in the plot) is created. These passages, also called “theoretical sentences” (Martinez and Scheffel, 2016, p. 105) or “essayistic passages” (Gittel, 2015), are often regarded as comments on the fictional world. Often, but not always, they include generalising statements. One example is found in *Geschichte des Agathon* (Wieland, 2012):

(1) *Der Gebrauch der Sprache hört auf, wenn sich die Seelen einander unmittelbar mitteilen, sich unmittelbar anschauen und berühren, und in einem Augenblick mehr empfinden, als die Zunge der Musen selbst in ganzen Jahren auszusprechen vermöchte*.
  ‘The use of language ceases when souls communicate directly with each other, look at each other and touch each other directly, and feel more in an instant than the tongue of the muses itself could express in whole years.'

This sentence, that clearly does not advance the plot, can be understood in different ways. On the one hand, it can be understood as a description of the characters' feelings in the fictional situation, and on the other hand, as an assertion about the connection of souls in general, even outside the fictional world. Both readings share that they are based on processes of reflection. We call those passages that represent a narrative pause and at the same time contain a generalisation, comment on the events of the story and/or suggest theses about the real world, “reflective passages” (see Gittel, 2022). We thus associate the phenomena generalisation, comment, and non-fictional speech with reflective passages.

### 2.2. Uncertain Attribution

It applies to both spoken and written language that an utterance can only be understood properly if it is clear who takes responsibility for the conveyed information. This may seem trivial for every-day language, since the interlocutors most often share direct contact in exchange of information. Nevertheless, even in every-day language, speakers use techniques to convey that someone else needs to be understood as the original speaker of an upcoming information, e.g., by using inquits like *she said*, or informal expressions such as *you won't believe what my daughter said to me*. However, in narrative fictional texts the communication framework is much more complex, since the communication is multi-layered. This is due to the fact that, by creating a fictional narrative, the author invites the reader to imagine a fictive narrator that tells the story. According to this (recently contested) narratological standard-view (e.g., Currie, 2010; Köppe and Stühring, 2011) there are two levels of communication: 1) a text-external level where an author communicates with the reader and 2) a text-internal, “fictional” level where a fictive narrator introduces certain characters which may communicate with the narrator or with other characters. The narrator is the speaker who reports the plot of a story, describes the scenery and provides background information on the characters. It can, but does not have to, appear explicitly in the text, e.g., by expressing itself in a self-referential manner or by making comments about the reported events. It can also appear in a personalised manner (e.g., as homodiegetic narrator) or as a named character in the story. In a story, the narrator and characters can be speakers, but the boundaries might be fluid:

(2) ≫*Es gibt keinen Sandmann, mein liebes Kind*≪*; erwiderte die Mutter*, ≫*wenn ich sage, der Sandmann kommt, so will das nur hei*β*en, ihr seid schläfrig und könnt die Augen nicht offen behalten, als hätte man euch Sand hineingestreut*.≪ (Hoffmann, 2012)
  “‘There is no sandman, my dear child” replied the mother, “when I say that the sandman is coming, it only means that you are sleepy and cannot keep your eyes open, as if sand had been put into them.”'

For the introductory verbum dicendi (*erwiderte* ‘replied'), the narrator is the speaker, reporting what the character (the mother) was doing—in this case, that the mother said something. The direct speech itself is character-attributed, since the mother is the speaker and the narrator does not intervene with her speech here—which is indicated by the quotation marks. Whenever a character is speaking directly, we define the character as the attributable speaker. However, it is important to note that certain literary storytelling techniques qualify automatically as character-attributed, e.g., inner monologues or streams of consciousness. In the next example, on the other hand, the narrator is the speaker by reproducing the character's speech indirectly:

(3) *Stechlins Eintritt ins Regiment fiel so ziemlich mit dem Regierungsantritt Friedrich Wilhelms IV. zusammen, und wenn er dessen erwähnte, so hob er, sich selbst persiflierend, gerne hervor*, ≫*daβ alles Groβe seine Begleiterscheinungen habe*≪. (Fontane, 2012)
  ‘Stechlin's entry into the regiment pretty much coincided with Friedrich Wilhelm IV's accession to power, and when he mentioned it, he liked to point out, satirising himself, “that everything great has its side effects.”’

Therein, the speakers overlap: the character who said something and the narrator reformulating it. Whenever narrator and character overlap, we understand this as uncertain attribution. We find such combined attributions in story-telling techniques such as indirect speech, stream of consciousness, and free indirect discourse. Thereby, the reader is not confronted with pure character speech but with a version of it, which is impacted by the narrator's point of view.

Up to this point we were only confronted with the text-internal communication. Now, we are turning to the text-external communication. Let us take a look at example (1) of a reflective passage again which does not contain characters' speech. The content of the statement can be understood as a description of the fictional world. So in this understanding the narrator is the speaker who is conveying the information. Considering the story was invented by an author, the utterance might also be interpreted as a statement about the real world (of which the author is part of). In this case, not only characters of the fictional world would be described, but persons in general. If a reader understands the passage here as non-fictional assertive speech, we must assume that the information can no longer be attributed to the narrator alone, since the narrator is a construct and knows nothing about the real world. One might assume that only the author knows about the real world and therefore needs to be at least one of the associated speakers here.

So let us summarise: If more than one speaker is attributed for one passage, this indicates one of the following cases or a combination of both: 1) the content of the passage is either rendered and cannot be unambiguously attributed to the narrator or a character alone and 2) the passage leads the reader to believe that it conveys the author's assertions or hypotheses. We assume that the multi-layered communication of narrative texts, especially in reflective passages, is characterised by differing speakers and thus by uncertain attribution. We speak of uncertain attribution whenever it is unresolvable whether the speaker (i.e., character, narrator and in certain cases the author) is identifiable as the entity to whom the information can be unambiguously attributed. Presumptively, the attribution of text passages to authors is especially impacted by literary theoretical beliefs and world knowledge of the recipients, particularly knowledge about the author. This problem cannot be solved hermeneutically (Schmid, 2011, p. 131 f.), but will be investigated empirically through our annotation.

## 3. Materials

### 3.1. Corpus and Annotation Guidelines

We currently construct a diachronic corpus of German fictional literature from 1650 to 1950. As of now, we annotated 10 texts (2,701 sentences / 61,979 tokens). CATMA^1^ appeared to be most suitable for annotating fictional texts and became our tool of choice. In order to create a versatile dataset and save resources, we annotate only the beginning of every text (usually about the first 200 sentences).

In this study, we are interested in annotation disagreements that are grounded in the annotators' literary preferences and implicit beliefs. We hypothesise that this applies to attribution, as discussed in the previous section, but not necessarily to the annotation of comment, generalisation, or non-fictional speech. For these phenomena, annotation disagreements are more likely to arise from *textual* (e.g., syntactic or semantic) ambiguity or vagueness, which is typical for natural language. Attribution is context-sensitive but not restricted to certain types of text passages, and is thus best viewed as a second annotation dimension that can be combined with various phenomena^2^.

Our annotation procedure consists of three steps: 1. annotation of reflective passages, 2. creation of a gold standard, and 3. annotation of speaker attribution.

#### 3.1.1. Annotation of Reflective Passages

First the annotators identify the three phenomena generalisation, comment, and non-fictional speech, which we consider to be potential indicators of reflective passages (see section Reflective Passages). We developed detailed annotation guidelines for these three phenomena [see Dönicke et al. (2021) for generalisation, and our annotation guidelines in Barth et al. (2021) for the other phenomena], which require reflective passages to be annotated at the clause or supra-clause level. If it is appropriate based on the phenomenon, a passage can comprise a single clause, several clauses or sentences, or even whole paragraphs. Furthermore, passages might be nested or overlap as we will see below.

Our annotators are students with a background in German philology and literature. All of them have several months of experience in annotating for our project and can thus be seen as expert annotators. We have six annotators overall, which means two annotators per phenomenon for a text. Table 1 shows Fleiss' inter-annotator agreement coefficient κ (Fleiss et al., 2003) for all three phenomena and averaged over texts. All phenomena are, as typical for natural-language annotation tasks, affected by textual ambiguity and therefore to some degree subject to the reader's interpretation. For comment, this is even more the case than for the other two phenomena, which explains the only moderate agreement in the comment annotation whereas generalisation and non-fictional speech are annotated with substantial agreement. The comparatively high standard deviations presumably result from the varying complexity of the literary texts and the language variant in which a text is written; we also change the constellation of annotators for every text, which leads to variation in inter-annotator agreement.

**Table 1:** Clause-level Fleiss' κ for the phenomena marking reflective passages.

**Phenomenon**	**μ(κ)**	**σ(κ)**
Generalisation	0.69	0.20
Comment	0.47	0.16
Non-fictional speech	0.70	0.16

*μ is the average agreement over all texts; σ is the corresponding standard deviation*.

#### 3.1.2. Creation of a Gold Standard

Having verified that comment, generalisation, and non-fictional speech can indeed be identified with moderate to substantial agreement, we next create a gold standard for this annotation that will then serve as the input for the attribution annotation. The gold standard is created through an adjudication step in which two researchers inspect the annotated texts, focusing on the passages that were labelled by at least one annotator. The adjudicators discuss the annotations, aiming to identify one prevalent interpretation (or, in exceptional cases, mark passages as textually ambiguous if two interpretations are equally plausible), and create the gold standard annotation.

#### 3.1.3. Annotation of Speaker Attribution

Finally, the annotators attribute the identified segments of the gold standard passages, using our attribution categories (i.e., character, narrator, and/or author). Attribution annotation is also performed on clause-level. As we are interested in investigating annotator bias for this task, we provide annotation guidelines which give annotators a lot of freedom to use their own judgement when attributing a passage. Specifically, the guidelines (Barth et al., 2021) only specify how labels should be assigned given an annotator's interpretation but they do not provide guidance on how to arrive at an interpretation. We also do not create a gold standard for attribution as we are interested in variation not homogeneity.

Example (4) shows an annotation example from *Ein Kampf um Rom* (Dahn, 2012) with three overlapping reflective passages (in the gold standard): 
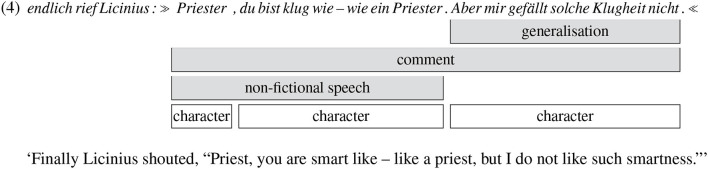


There is a generalisation passage covering one clause, a comment passage covering three clauses, and a passage of non-fictional speech covering two clauses^3^. One of our annotators assigned the attribution label “character” to all of the three involved clauses (Punctuation at span boundaries must not be annotated due to our guidelines). The first clause *endlich rief Licinius* ‘finally Licinius shouted' is not annotated with attribution because it is not part of a reflective passage.

As of now, the entire corpus contains 1,712 reflective passages, which consist of 3,565 unique clauses^4^.

### 3.2. Data Preparation

Since attribution labels are assigned on clause-level, we prepare our corpus data for a classification task which is to predict the labels for a specific clause in a certain context. As context of a clause, we use the sentence that contains the clause as well as its preceding and succeeding sentence. Since one and the same context possibly contains several annotated clauses, for which the annotators give individual attribution labels, the initial sample format is context ↦ {clause ↦ (annotator ↦ labels)}. To reduce the input size for the neural classifier (see section Method), we only keep contexts with a maximum of 100 tokens, totalling to 1,058 contexts (79% of all 1,340 unique contexts). We randomly split the contexts into a training set, a development set and a test set which have 80%, 10% and 10% of the data.

In a second step, we construct samples of the form (context, clause) ↦ {annotator ↦ labels}. Transforming the data into this format increases the sample size since each context contributes as many samples as it has annotated clauses (see Table 2). For example, the first two annotated clauses in (4) appear within the same sentence and thus have the same context, which happens to be the entire text of the example (one additional preceding and succeeding sentence). Therefore, this context contributes two samples. The third annotated clause in (4) receives another context with the context window moved one sentence to the right. The training, development and test sets then contain 1,897 (78%), 267 (11%), and 254 (11%) clauses, respectively.

**Table 2:** Number of samples in training, development and test set.

**Sample format**	**Train set**	**Dev set**	**Test set**
context ↦ {clause ↦ (annotator ↦ labels)}	846	106	106
(context, clause) ↦ {annotator ↦ labels}	1,897	267	254
(context, clause, annotator) ↦ labels	11,382	1,602	1,524

Finally, we construct samples of the form (context, clause, annotator) ↦ labels, which increases the sample size by a factor of 6 (i.e., the number of annotators). The distribution of labels in the data is shown in Table 3.

**Table 3:** Label distributions in training, development and test set.

	**Character**	**Narrator**	**Author**
Train set	0.46	0.61	0.20
Dev set	0.44	0.63	0.24
Test set	0.47	0.60	0.18

### 3.3. Questionnaire

To determine correlations between literary beliefs and annotators' annotation behaviour, we constructed a questionnaire which focuses on revealing biases regarding literary beliefs. The questionnaire is composed of two parts: part A consists of the Literary Response Questionnaire (LRQ) from Miall and Kuiken (1995), a psychological questionnaire that captures seven different aspects of readers' orientation towards literary text. Questions in it refer to insight, empathy, imagery vividness, leisure escape, concern with the author, story-driven reading, and rejection of literary values. The LRQ is supplemented by part B, which we developed specifically for this work. We aim to capture biases towards literary texts, especially with respect to all attribution categories (character, narrator, author). We assume that literary beliefs have a great influence on annotation behaviour because, for example, annotating the author as a speaker is more or less likely depending on literary background. Therefore, part B of the questionnaire asks about (implicit) theoretical literary beliefs related to the narrator, the author as a decisive criterion for reading a text, the text as mean of communication with the author or authorial intention, narrator theories, implicit authorial instances, the relationship between the author and characters, and the relationship between the author and the narrator.

Since the focus of our research lies on German literature and our annotators are all German native speakers, we prepared part B of the questionnaire in German and also translated part A into German (the complete questionnaire is contained in the Supplementary Materials). The entire questionnaire consists of 14 thematically coherent groups of questions (A1,..., A7; B1,..., B7) and a group of filler questions (B8), with a total number of 94 questions. When the questionnaire was given to the annotators, questions appeared in randomised order; at first the questions from part A, followed by the questions from part B, but without letting the participants know when part B started. All answers are given on a Likert scale from 1 (“I totally disagree”) to 5 (“I fully agree”).

## 4. Methods and Results

### 4.1. Bias Analysis

#### 4.1.1. Method

Our first experiment aims at finding correlations between implicit literary convictions of the annotators and annotations that they produce. Therefore, we randomly split the 1,897 training clauses into 18 batches of size 100 (omitting 97 samples) and compute the distribution of the labels for each batch–annotator pair (see Table 4). We then extend each row with the questionnaire answers of the corresponding annotator as well as the mean values for each question group. We use this data to calculate correlations for each label and each question or question group, e.g., by calculating the correlation between the columns for the label “character” and the question A1F1. For the computation, we use Pearson's correlation coefficient.

**Table 4:** Excerpt from the data for the correlation experiment: Label distributions of a specific annotator in a specific batch, and questionnaire answers of the corresponding annotator.

		**Labels**			**Questions**			**Group means**		
**Batch**	**Annotator**	**Character**	**Narrator**	**Author**	**A1F1**	**…**	**B8F5**	**A1**	**…**	**B7**
#1	An1	0.45	0.60	0.24	4	…	3	3.8	…	4.0
#1	An2	0.56	0.50	0.20	2	…	2	3.0	…	5.0
⋮	⋮	⋮	⋮	⋮	⋮	⋱	⋮	⋮	⋱	⋮
#18	An5	0.45	0.56	0.02	4	…	2	3.2	…	3.7
#18	An6	0.55	0.59	0.04	4	…	2	3.0	…	4.0

#### 4.1.2. Results

Table 5 shows Pearson correlation coefficients between labels and features (i.e., questions or question groups), calculated over all 18 × 6 batch–annotator combinations. Since we are interested in features that show significant differences between annotators, we exclude features whose answers show a low variance among annotators. For example, question A1F4 received the answers (4, 2, 4, 4, 4, 4) by our annotators, which corresponds to a variance of 0.67. We only keep features with a variance greater than 0.8, where 0.8 is the variance of the combinations {(1, 1, 2, 2, 3, 3), (2, 2, 3, 3, 4, 4), (3, 3, 4, 4, 5, 5)} (and their permutations).

**Table 5:** Correlations between labels and questions with an absolute value of 0.5 and higher.

**Label**	**Question**	**Question group**	**Variance**	**Correlation coefficient**	**Rank**
Character	A3F5	Imagery	1.81	−0.62	
Character	A5F9	Concern with author	1.58	−0.59	
Character	A2F4	Empathy	1.47	−0.57	
Character	B5F1	Implicit author	0.81	−0.57	
Character	A1F5	Insight	0.89	−0.56	
Character	A7F4	Rejection of literary values	1.14	0.56	
Character	A1F10	Insight	1.22	−0.54	
Character	A7F8	Rejection of literary values	1.47	0.52	
Narrator	A5F2	Concern with author	0.89	0.62	10
Narrator	B3F1	Text as message of the author	1.14	0.56	
Narrator	A1F13	Insight	0.81	0.51	
Narrator	B1F3	Preferences for manifest narrators	0.81	0.50	
Author	A5F5	Concern with author	1.89	0.89	1
Author	A4F6	Leisure Escape	1.14	0.83	2
Author	A2F5	Empathy	1.22	0.82	3
Author	A3F3	Imagery	1.22	−0.79	4
Author	B5F3	Implicit author	0.92	0.71	5
Author	B1F3	Preferences for manifest narrators	0.81	0.67	6
Author	B1F1	Preferences for manifest narrators	0.81	0.66	7
Author	A7F8	Rejection of literary values	1.47	0.65	8
Author	A1F13	Insight	0.81	0.63	9
Author	A6F3	Story-driven reading	1.56	0.62	
Author	A4F3	Leisure escape	0.89	0.61	
Author	A4F2	Leisure escape	1.56	0.58	
Author	A6F8	Story-driven reading	0.92	0.54	

*The rank column indicates the overall top-10 features*.

Correlations with an absolute value ≥ 0.5 can be considered as high. It can be seen that the label “author” shows a high correlation with more questions (13 questions) than the label “character” (8 questions), which in turn shows a high correlation with more questions than the label “narrator” (4 questions). This is in line with what we would expect: Whether the author is interpreted as taking responsibility for the information conveyed in a passage is typically not explicitly signalled in the text and, as such, it leaves much room for diverging annotation decisions based on the annotator's preferences and beliefs. Neither any of the filler questions (from B8) nor any of the question-group means reaches high correlation coefficients with any label.

The top-10 questions with the highest correlations are taken as features in some of the classification experiments. Almost all of these features correlate with the label “author” and only one with the label “narrator” (A2F5), scoring only marginally higher than A3F5 for “character” and A6F3 for “author”. To investigate the impact of each annotator on the correlation results, we calculate correlation coefficients for six groups, where one of the annotators is excluded (see Table 6). One can observe that if one of the annotators is missing, the top-10 features notably change. They change most if annotator 5 is excluded. Among the top-10 features, questions A5F5 and A2F5 remain consistent in all constellations, questions A4F6 and B5F3 mostly behave equally, while A1F13 and A5F2 are less consistent and are mostly removed from the top-10 if one of the annotators is excluded.

**Table 6:** Most correlated features (questions) when one of the annotators is excluded: “+” if the feature remains among the top-10 correlated features, “–” if it gets removed.

**Excluded**	**Overall top-10 features**									
**Annotator**	**A5F5**	**A4F6**	**A2F5**	**A3F3**	**B5F3**	**B1F3**	**B1F1**	**A7F8**	**A1F13**	**A5F2**
An1	+	+	+	+	+	+	–	+	–	–
An2	+	+	+	+	+	+	–	–	–	–
An3	+	+	+	+	+	+	+	–	–	–
An4	+	+	+	+	+	–	+	+	–	–
An5	+	–	+	–	–	–	–	+	–	–
An6	+	+	+	–	+	+	+	–	+	+

Table 7 shows the top-10 correlating features and the individual answers of the annotators. One can see that annotator 5 shows an extreme value (i.e., single-lowest or single-highest value) for 8 of the 10 questions. This explains why there is the most significant change when excluding annotator 5 in Table 6: in 7 cases, the feature does not pass the variance filter anymore.

**Table 7:** Overall top-10 questions with the answers given by the annotators.

**Rank**	**Question**	**Text (English version)**	**Answers**					
			**An1**	**An2**	**An3**	**An4**	**An5**	**An6**
1	A5F5	When reading I usually try to identify an author's distinctive themes.	5	4	3	4	1	5
2	A4F6	While reading I completely forget what time it is.	4	4	3	3	1	4
3	A2F5	I actively try to project myself into the role of fictional characters, almost as if I were preparing to act in a play.	4	3	2	2	1	4
4	A3F3	I sometimes think I could draw a map of the places I have read about in a work of fiction.	3	2	3	1	4	1
5	B5F3	When reading a literary text, I bring to my mind that my idea of the author does not necessarily resemble the real author.	4	3	2	2	1	3
6	B1F3	I like to read novels in which the narrator often comes to the fore and the story takes a back seat.	5	4	4	4	2	4
7	B1F1	I like to read novels that have a first-person narrator.	4	4	4	5	2	4
8	A7F8	Works of literature often seem to make the issues of life more complicated than they actually are.	1	3	1	1	1	4
9	A1F13	Literature often gives special emphasis to those things that make a moral point.	4	4	2	4	2	3
10	A5F2	In reading I like to focus on what is distinctive about the author's style.	5	3	3	4	2	3

Overall, our results suggest that there is indeed a certain amount of interdependence between annotator's beliefs and preferences (as captured by the questionnaire) and their attribution choices. Some findings seem plausible, others are more difficult to explain. For example, features like A5F5 which focus on the author are likely to have an influence on the annotation behaviour; while one cannot think of a straightforward connection between features like A4F6 and the annotation.

Given that we only have six annotators, we refrain from carrying out a deeper analysis at this point. However, we will make use of the findings when selecting features for the machine learning experiments in the following section.

### 4.2. Neural Attribution Classification

#### 4.2.1. Method

**Figure 1:**
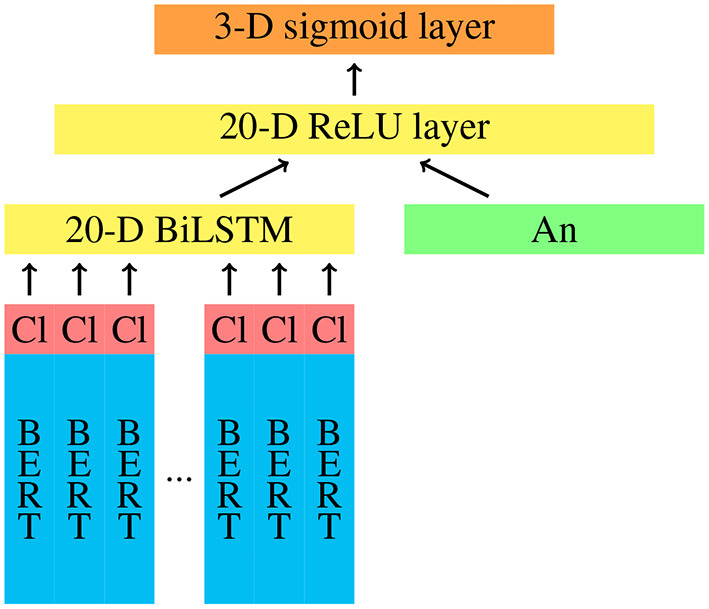
Architecture of the neural network with annotator encoding (An), BERT embeddings, and clause encodings (Cl).

We implement a neural classifier using Keras^5^, which is trained in different conditions, yielding several individual models. The architecture of our models is shown in Figure 1. The context tokens of a sample are vectorised with a pretrained German BERT model^6^. To encode the clause of the sample, we add an additional dimension to the BERT embeddings, which has a value of 1 for tokens that belong to the clause and a value of 0 for tokens that constitute the context. The sequence of (768+1)-dimensional embeddings is fed to a 20-dimensional BiLSTM layer. Here, we use a dropout rate of 0.2 for the inputs and no dropout for the recurrent state. The output of the BiLSTM is then concatenated with a feature vector that encodes the annotator of the sample and depends on the training condition (see below). The concatenated input is fed to a 20-dimensional dense layer with ReLU activation and an L2 regularisation factor of 10^−4^. Since the network should learn a multi-class multi-label classification, we use sigmoid activation in the output layer and binary cross-entropy as loss function. We use the Adam optimisation with a learning rate of 10^−4^. Each model is trained with a batch size of 6 for 30 epochs^7^.

First of all, we distinguish three training-set conditions (keywords for later reference are bold):

1. We train six individual models for the **unaggregated** training sets of the individual annotators (1,897 training samples each). Each individual model should learn to make predictions for one specific annotator.
2. We train one model for the **aggregated** training sets of all annotators (11,382 training samples). This model should learn to make predictions depending on a specified annotator.
3. We train six models for the aggregated training sets of all but one annotators, i.e., one annotator is excluded during training (9,485 training samples each). These models should be tested in a **cross-validated** fashion to evaluate the predictions for an unseen annotator.

When training on unaggregated sets, no annotator encoding is required since all labels belong to the same annotator. In the other two conditions, we experiment with different annotator encodings:

1. We use **no encodings** at all (as for the unaggregated training sets). This leads to possibly conflicting samples, since there could be samples with identical input (clause and context but no annotator) but different labels (from different annotators).
2. We use 6-dimensional **one-hot** encodings for the annotators.
3. We use 108-dimensional **questionnaire** encodings, combining the 94 values received from the questionnaire and the 14 mean values for every question group. Hereby, the values of the questionnaire, which originally lie in [1, 5], are shifted to [−2, 2], so that the value 0 indicates neutrality.
4. We only use a top-10 **selection** of those questionnaire features that show the highest correlation with any label in the training set (see section Bias Analysis) for the correlation results).
5. For the cross-validated condition, we recalculate the correlations on the training set without the excluded annotator and use an adjusted selection* of the top-10 correlating features.

When it comes to prediction, we investigate two conditions for the aggregated models:

1. All annotator encodings are **given** to the model as in training. In this condition, the model should predict the labels of a specified annotator.
2. All annotator encodings are **zeroed**, i.e., the model is only given zero vectors. Here, the model should predict the labels that a common-sense or neutral annotator would assign.

#### 4.2.2. Results

We use binary (i.e., micro-averaged) accuracy (Acc) as evaluation measure for our models. Since the labels in our dataset are not distributed uniformly, we calculate the majority baseline for comparison. The majority baseline is the maximal accuracy that a model could achieve if it would predict the same output for all test samples. As secondary evaluation measures, we compute micro-averaged (Mic) and macro-averaged (Mac) f-score.

In a first experiment, we test whether the context representations are expressive enough to learn attribution categories at all. For this, we train a model with one-hot annotator encodings. Table 8 shows that the model correctly predicts 96% of the labels from seen samples (i.e., the training set). As typical for NLP tasks, the accuracy on unseen samples is lower, namely 85% on the development and 88% on the test set. However, this still outperforms the majority baseline by ≥20%.

**Table 8:** Performances on the aggregated training, development and test sets after training on the aggregated training set with one-hot annotator encodings.

	**Train set**			**Dev set**			**Test set**		
**Annotator encoding**	**Acc**	**Mic**	**Mac**	**Acc**	**Mic**	**Mac**	**Acc**	**Mic**	**Mac**
Majority baseline	0.65	–	–	0.65	–	–	0.65	–	–
One-hot given	0.96	0.95	0.93	0.85	0.83	0.81	0.88	0.85	0.80

In a second set of experiments, we test whether the classifier captures similarities between annotators. A model that is trained on one of the unaggregated training sets for a specific annotator should achieve the highest accuracy on the corresponding unaggregated test set of the same annotator. Indeed, Table 9 shows that the highest accuracy for each annotator is achieved on the corresponding test set.

**Table 9:** Accuracies on the unaggregated test sets after training on the unaggregated training sets.

	**Test set**					
**Train set**	**An1**	**An2**	**An3**	**An4**	**An5**	**An6**
An1	**0.87**	0.78	0.76	0.85	0.76	0.72
An2	0.77	**0.87**	0.77	0.75	0.76	0.79
An3	0.75	0.78	**0.86**	0.77	0.86	0.77
An4	0.85	0.77	0.75	**0.86**	0.78	0.75
An5	0.77	0.80	0.81	0.78	**0.87**	0.74
An6	0.78	0.83	0.78	0.79	0.76	**0.88**

*Row names and column names represent annotators. Bold values mark accuracies for each test set after training on the corresponding training set*.

In more general words, each model should achieve higher accuracies on test sets of annotators that are more similar to the annotator of the training set. The similarity of two annotators can be measured with Krippendorff's inter-annotator-agreement coefficient α (Krippendorff, 2018, pp. 221–250), using the MASI distance (Passonneau, 2006) for multi-label annotations. Table 10 shows inter-annotator agreements for all pairs of annotators. The Pearson correlation between Tables 9 and 10 is 0.88, indicating that the more similar the test annotator is to the training annotator, the more accurate are the model's predictions.

**Table 10:** Pairwise Krippendorff's α on the training set.

	**Annotator**					
**Annotator**	**An1**	**An2**	**An3**	**An4**	**An5**	**An6**
An1	1.00	0.68	0.64	0.90	0.56	0.67
An2	0.68	1.00	0.62	0.67	0.62	0.72
An3	0.64	0.62	1.00	0.63	0.73	0.60
An4	0.90	0.67	0.63	1.00	0.58	0.68
An5	0.56	0.62	0.73	0.58	1.00	0.50
An6	0.67	0.72	0.60	0.68	0.50	1.00

*Row names and column names represent annotators. Note that Krippendorff's α is symmetric*.

**Figure 2:**
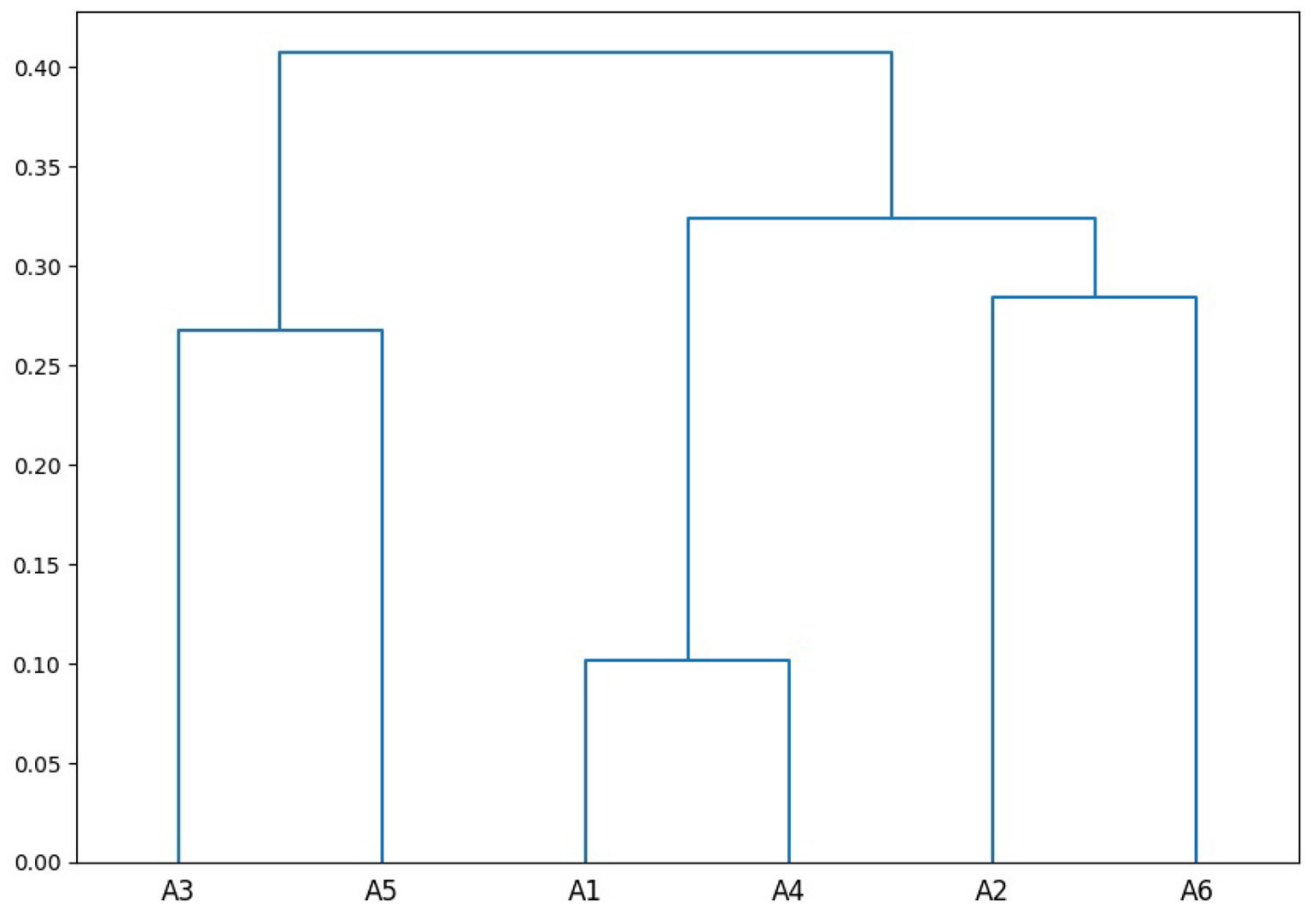
Clustering of annotators using the pairwise disagreement (1 − α) on the training set.

Figure 2 further shows a hierarchical clustering of all annotators on the training set using 1 − α as distance metric and the unweighted pair group method with arithmetic mean (UPGMA; Sokal et al., 1958) as clustering method. We can see that there are sub-groups of annotators, e.g., annotator 1 and annotator 4 annotated similarly (α = 0.90). Consequently, a model trained on annotator 1's training set makes the second-best predictions on annotator 4's test set (the best predictions still makes a model trained on annotator 4's training set), and vice versa.

The main set of experiments compares the performances when using different annotator encodings. The results are shown in Table 11. As we have already seen in Table 8, a model with one-hot encodings achieves an accuracy of 88% when annotator encodings are given during prediction. When they are zeroed, the accuracy drops to 84%, which is the same accuracy as a model with no annotator encodings achieves. This indicates that the one-hot model correctly interprets the zero vector as common-sense annotator, although it has not seen any such vector during training. The models with questionnaire and selection encodings achieve accuracies of 87 and 88%, respectively, when annotator encodings are given but behave quite differently when the encodings are zeroed: The accuracy for the questionnaire model drops to 77% whereas the accuracy for the selection model only drops to 84%. We theorise that the differences in performance can be explained as follows: The 108-dimensional questionnaire encodings presumably contain a lot of dimensions with lower variance between the annotators, making the questionnaire encodings more similar to each other than the selection encodings. This makes the encodings less distinctive which could explain the lower performance of 87% when compared to that of e.g., one-hot encodings. Furthermore, the zero vector represents a neutral annotator, i.e., an annotator that would answer all questions in the questionnaire with “neither agree nor disagree”; this is not necessarily the average or common-sense of our six annotators. The selection encodings, on the other hand, are based on distinctive features, i.e., features that are low (< 0) for some annotators and high (> 0) for other annotators. The zero vector then represents a non-extreme annotator that is likely to be similar to the average of our six annotators. Hence, the selection encodings perform as well as one-hot encodings.

**Table 11:** Performances on the aggregated test set after training on the aggregated training set with different annotator encodings, and with given or zeroed annotator encodings at prediction time.

**Annotator encoding**		**Acc**	**Mic**	**Mac**
No encoding	–	0.84	0.81	0.74
One-hot	Given	0.88	0.85	0.80
	Zeroed	0.84	0.81	0.75
Questionnaire	Given	0.87	0.84	0.78
	Zeroed	0.77	0.73	0.66
Selection	Given	0.88	0.85	0.79
	Zeroed	0.84	0.81	0.73

In a classification scenario where all annotators are seen in training, one-hot encodings would be sufficient as encoding of choice. In a last experiment, we investigate the effect of the type of annotator encoding if we want to make predictions for an annotator unseen in training. Table 12 shows cross-validation results for questionnaire and selection encodings, with no encoding as baseline. The baseline accuracies range from 77 to 84% with an average of 81%. This result cannot be outperformed by questionnaire encodings which only show a greater standard deviation with accuracies ranging from 76 to 85%. Selection encodings, on the other hand, are able to improve the performance to accuracies of 82% in average. However, the selection is determined for all annotators, including the test annotator, in the first place, whereas in a real-world scenario one could determine the selection* on the training annotators' annotations only. When doing so, the accuracy decreases to the level of the baseline. Interestingly, the results with no encodings show a parallel to that with selection* encodings, and the results with questionnaire encodings show a parallel to that with selection encodings. We do not have a straightforward explanation for this observation at hand. As a conclusion of this experiment, one can say that none of the possible encodings (no encoding, questionnaire, selection*) is more suitable than the others for predicting the labels of an unseen annotator.

**Table 12:** Cross-validation performances on the unaggregated test sets after training on the aggregated training sets.

**Annotator encoding**		**An1**	**An2**	**An3**	**An4**	**An5**	**An6**	**μ**	**σ**
No encoding	–	0.83	0.84	0.81	0.81	0.82	0.77	0.81	0.02
Questionnaire	Given	0.83	0.82	0.76	0.85	0.81	0.81	0.81	0.03
Selection	Given	0.83	0.85	0.76	0.81	0.85	0.81	0.82	0.03
Selection*	Given	0.82	0.83	0.82	0.81	0.81	0.77	0.81	0.02

*The training samples of the test annotator (column name) were not included in the aggregated training set. The right-most columns show average (μ) and standard deviations (σ) for each six runs*.

We provide more detailed classification results, e.g., f-scores for some of the mentioned experiments or performances of all models on the unaggregated test sets, in the Supplementary Materials.

## 5. Discussion

In recent years, more and more researchers have begun to study annotator bias. Most of these studies are focused on the effect of bias on the quality of annotated data. This is particularly important if the annotations are done in a crowd-sourcing scenario, where the identification of spammers is crucial (cf. Soberón et al., 2013; Paun et al., 2018). Other studies looked into bias effects that may arise from how the annotation task is formulated, especially in the areas of natural language inference and generation (Amidei et al., 2018; Gururangan et al., 2018; Tsuchiya, 2018). An area where annotator bias is particularly relevant is hate speech detection and several papers have looked into different types of bias. For instance, Kuwatly et al. (2020) investigate how different user demographics influence hate speech annotations, while Wich et al. (2020) look at the impact of political biases.

Annotator bias that arises when annotating linguistic phenomena has also received a lot of attention (Morris and Hirst, 2004; Morris, 2010; Rohde et al., 2016; Scholman and Demberg, 2017). It has been shown that for linguistic annotations, annotator certainty is often not correlated with annotation variation (Poesio and Artstein, 2005; Nedoluzhko and Mírovský, 2013; Andresen et al., 2020), indicating that disagreements between annotators may be influenced by annotator preferences. Annotation bias in literature has only rarely been discussed, which is surprising given that the literature analysis is often seen as inherently open to interpretation and divergence of opinion (Hammond et al., 2013). One exception is a study by Gius and Jacke (2017), who classify annotation disagreements into four classes: misinterpretations, deficient category definitions, categories which depend on preliminary analyses, and textual ambiguities or polyvalence. Only the latter are seen as adequate reasons for disagreement. From an application perspective, the work that comes closest to our present study is Hammond et al. (2013), which is concerned with tracking attribution ambiguity for free indirect discourse in Virgina Woolf's *To the lighthouse* (1927). However, that paper describes work in progress and no results are given for the annotation experiment. Details on the planned machine-learning study are also sketchy.

From a machine-learning and computational-modelling perspective, several studies have shown that annotation bias can harm the performance of classifiers trained on the data (cf. Gururangan et al., 2018; Tsuchiya, 2018) and that annotator information can improve performance. For example, working on hate speech detection, Akhtar et al. (2020) divide annotators into two groups, building on their earlier work on measuring polarisation in hate speech annotation (Akhtar et al., 2019). They train a classifier for each annotator group and also build an ensemble classifier, which labels an instance as hate speech whenever one of the individual classifiers did so. They find that the latter outperforms the former. Working on various natural language understanding tasks, Geva et al. (2019) take a slightly different approach and—similarly to us—include identifiers for individual annotators in the input feature vector for a neural-network architecture. They show that this improves performance compared to an annotator-agnostic classifier but the model is not able to generalise to unseen annotators. Note, however, that Geva et al.'s data comes from natural language inference and question answering, where annotations are not simple labels but complete sentences which will carry inevitably a stronger annotator signal.

In this study, we combine previous research on annotator bias (and the reasons for it) with work on modelling bias computationally and apply it to a domain in which annotation bias has so far been under-researched, namely literature.

Specifically, we address the task of automatic speaker attribution in fictional narrative texts. This is a challenging task as the attribution of one or several speakers to a text passage does not solely depend on the text itself but also on the person who reads and interprets it—which constitutes the phenomenon of uncertain attribution. In consequence, a classifier has to be trained on both text representations and features that capture a reader's bias to become fully capable of the task. The first question which we followed was to find such bias features by correlating the attribution annotations of six annotators with the answers they gave in an extended version of Miall and Kuiken (1995)'s Literary Response Questionnaire. We found that the attribution category “author” is, in comparison to “narrator” and “character”, subject to the most bias features that were tested. Since narrator and character speech is at least partially marked at the surface text, whereas author speech requires an additional interpretation (except for passages in a preface or the like), we claim (following e.g., Schönert, 2014) that this category is more subjective than the other two, which is underpinned by the correlation results.

We experimented with a neural classifier that is trained on pairs of text representation and a bias feature vector, and thus can learn to make biased predictions. We showed 1) that the neural architecture is capable of learning similarities and differences between sub-groups of annotators, and 2) that a single model can learn to produce accurate predictions for individual annotators as well as 3) average or common-sense predictions for an unspecified or unseen annotator. Although the nature of speaker attribution does not suggest to create a gold-standard annotation, we are confident that one can use these common-sense predictions for follow-up applications and analyses. For example, one could automatically label an extended diachronic text corpus with speaker attribution and analyse the distribution of the three categories over time.

In future work, we plan to extend the set of annotators. In the present study, we only looked at six annotators, who can be seen as expert annotators in that they were all (advanced) students of German literature and, moreover, had 6 months to a whole year of experience at annotating the literary categories in our project. In a follow-up study we intend to also look at lay annotators, for example recruited through crowd sourcing. Moving to lay annotators would allow us to take into account a larger group of annotators and thereby hopefully shed more light on the effect of literary preferences on annotation decisions. Furthermore, it would enable us to compare two different groups of annotators and investigate, for example, whether annotator bias tends to be stronger for one of the groups.

## Data Availability Statement

The datasets and scripts generated for this study can be found in the repository https://gitlab.gwdg.de/mona/neural-attribution.

## Ethics Statement

Ethical review and approval was not required for the study on human participants in accordance with the local legislation and institutional requirements. The patients/participants provided their written informed consent to participate in this study.

## Author Contributions

The questionnaire was translated and extended by TD, BG, AW, and FB. TD and HV conducted the questionnaire-correlation experiment. CS and TD planned and designed the classification experiments. TD supervised the questionnaire experiment, prepared the corpus and questionnaire data, implemented the neural networks, and conducted the classification experiments. AH, BG, LG, AW, and FB developed annotation guidelines for attribution. AW supervised the annotation process. LG, AW, FB, HV, and TD created the gold standard for reflective passages. All authors together developed and discussed the general outline of the study as well as the conceptualization and formalization of the relevant literary phenomena and wrote sections of the manuscript, contributed to manuscript revision, read, and approved the submitted version.

## Funding

This work was funded by Volkswagen Foundation (TD, AW, LG, BG, AH, and CS), by the Deutsche Forschungsgemeinschaft (DFG, German Research Foundation) – 424264086 (HV, FB, BG, AH, and CS), and by Niedersächsisches Vorab.

## Conflict of Interest

The authors declare that the research was conducted in the absence of any commercial or financial relationships that could be construed as a potential conflict of interest.

## Publisher's Note

All claims expressed in this article are solely those of the authors and do not necessarily represent those of their affiliated organizations, or those of the publisher, the editors and the reviewers. Any product that may be evaluated in this article, or claim that may be made by its manufacturer, is not guaranteed or endorsed by the publisher.

## Footnotes

^1^ https://catma.de/

^2^We hypothesise that the annotation decisions for attribution are largely independent from the exact nature of the phenomenon to be attributed, i.e., when deciding whether the content of a text passage should be attributed to a character, the narrator, or the author it does not matter too much whether the attributable phenomenon is “comment”, “non-fictional speech”, or “generalisation”.

^3^Clause segmentation is performed with the clausizer presented in Dönicke (2020). The manually created clause-level annotations are then automatically mapped to the detected clauses.

^4^The corpus and annotation guidelines are published in Barth et al. (2021).

^5^ https://keras.io

^6^ https://huggingface.co/dbmdz/bert-base-german-cased

^7^We used the development set for manual tuning of the parameters described here.
