# Supplementary material for: Modelling Speaker Attribution in Narrative Texts With Biased and Bias-Adjustable Neural Networks
Source: Front Artif Intell. 2022 Feb 3;4:725321. doi: 10.3389/frai.2021.725321 (PMC8851310; doi:10.3389/frai.2021.725321)
Supplement: Supplementary file 1 [file Data_Sheet_1.PDF]

Supplementary Material

1 CLASSIFICATION RESULTS

			test set																				
			unaggregated																		aggregated /		
			An1			An2			An3			An4			An5			An6			average		
train set	annotator encoding		Acc	Mic	Mac	Acc	Mic	Mac	Acc	Mic	Mac	Acc	Mic	Mac	Acc	Mic	Mac	Acc	Mic	Mac	Acc	Mic	Mac
majority baseline			.70	–	–	.63	–	–	.65	–	–	.69	–	–	.68	–	–	.59	–	–	.65	–	–
unaggregated	no encoding	An1	.87	.85	.80	.78	.75	.69	.76	.71	.59	.85	.82	.76	.76	.69	.49	.72	.70	.65	.79	.75	.68
		An2	.77	.74	.69	.87	.85	.82	.77	.72	.62	.75	.72	.67	.76	.70	.53	.79	.78	.77	.78	.75	.70
		An3	.75	.70	.57	.78	.73	.60	.86	.82	.69	.77	.72	.59	.86	.81	.55	.77	.73	.60	.80	.75	.60
		An4	.85	.82	.77	.77	.73	.69	.75	.69	.56	.86	.83	.76	.78	.72	.52	.75	.72	.67	.79	.75	.68
		An5	.77	.70	.50	.80	.74	.54	.81	.75	.52	.78	.72	.51	.87	.82	.54	.74	.68	.53	.79	.73	.52
		An6	.78	.76	.73	.83	.82	.79	.78	.75	.66	.79	.77	.73	.76	.70	.56	.88	.87	.87	.80	.78	.74
aggregated	no encoding	–	.85	.81	.79	.87	.84	.78	.84	.80	.67	.84	.81	.77	.83	.78	.56	.83	.81	.76	.84	.81	.74
	one-hot	given	.90	.88	.83	.87	.85	.80	.88	.84	.67	.89	.87	.81	.89	.84	.57	.83	.82	.80	.88	.85	.80
		zeroed	.86	.83	.80	.86	.83	.78	.84	.80	.67	.86	.83	.78	.83	.78	.57	.82	.80	.77	.84	.81	.75
	questionnaire	given	.88	.85	.78	.90	.88	.85	.88	.84	.70	.85	.82	.74	.90	.85	.57	.79	.79	.76	.87	.84	.78
		zeroed	.84	.81	.75	.80	.76	.74	.73	.66	.53	.82	.79	.71	.73	.65	.46	.72	.70	.67	.77	.73	.66
	selection	given	.89	.87	.82	.88	.86	.82	.87	.82	.67	.88	.86	.79	.90	.85	.57	.84	.83	.80	.88	.85	.79
		zeroed	.85	.81	.78	.87	.84	.78	.85	.81	.69	.83	.79	.74	.85	.79	.56	.82	.79	.74	.84	.81	.73
cross-validated	no encoding	–	.83	.80	.75	.84	.81	.77	.81	.76	.62	.81	.77	.66	.82	.76	.56	.77	.74	.67	.81	–	–
	questionnaire	given	.83	.79	.64	.82	.80	.76	.76	.72	.60	.85	.83	.76	.81	.73	.51	.81	.78	.75	.81	–	–
	selection	given	.83	.80	.75	.85	.83	.80	.76	.68	.52	.81	.77	.71	.85	.79	.53	.81	.80	.80	.82	–	–
	selection*	given	.82	.78	.65	.83	.80	.74	.82	.78	.66	.81	.76	.55	.81	.75	.56	.77	.74	.70	.81	–	–

Table S1. Performances in all experiments. The left-most column corresponds to the training-set condition (unaggregated/aggregated/cross-validated) in which a model was trained. The second column shows the annotator encoding that was used for training. The third column shows either the annotator whose training set was used in training for the unaggregated condition, or whether the annotator encoding was given or zeroed when testing the model for the other conditions. The main columns correspond to the test sets on which a model was evaluated, comprising the six unaggregated test sets and the aggregated test set. Sub-columns show values for binary accuracy (Acc), micro-averaged f-score (Mic) and macro-averaged f-score (Mac). In the cross-validated condition, there is no aggregated test set; instead the average of the accuracies on the unaggregated test sets is shown. The first row below the header shows the majority baseline for each test set.

2 QUESTIONNAIRE

Code	German	English
Part A	***Translation used in this work***	***Original used in Miall and Kuiken (1995)***
A1	**Erkenntnis**	**Insight**
A1F1	Das Lesen von Literatur macht mich empfindsam für Aspekte meines Lebens, die ich normalerweise nicht beachte.	Reading literature makes me sensitive to aspects of my life that I usually ignore.
A1F2	In literarischen Werken erkenne ich manchmal Gefühle, die ich in meinem täglichen Leben nicht wahrgenommen habe.	In literature I sometimes recognize feelings that I have overlooked during my daily life.
A1F3	Ich erlebe meine Schwächen oft durch Figuren in literarischen Texten.	I often find my shortcomings explored through characters in literary texts.
A1F4	Ich habe den Eindruck, dass Literatur mir dabei hilft, das Leben von Menschen zu verstehen, die anders sind als ich.	I find that literature helps me to understand the lives of people that differ from myself.

A1F5	Das Lesen von Literatur gibt mir oft Einblicke in die Natur von Menschen und Ereignissen in meiner Welt.	Reading literature often gives me insights into the nature of people and events in my world.
A1F6	Ich sehe oft Ähnlichkeiten zwischen Ereignissen in der Literatur und Ereignissen in meinem eigenen Leben.	I often see similarities between events in literature and events in my own life.
A1F7	Ich erlebe meine eigenen Beweggründe oft durch Figuren in literarischen Texten.	I often find my own motives being explored through characters in literary texts.
A1F8	Ich habe den Eindruck, dass gewisse literarische Werke mir helfen, meine negativeren Gefühle zu verstehen.	I find that certain literary works help me to understand my more negative feelings.
A1F9	Literatur ermöglicht es einem, Menschen zu verstehen, die man im normalen Leben wahrscheinlich übersehen würde.	Literature enables you to understand people that you'd probably disregard in normal life.
A1F10	Ich bemerke manchmal, dass das Lesen eines literarischen Textes dafür sorgt, dass ich meine Lebensweise ändern will.	I sometimes find that reading a literary text makes me feel like changing the way I live.
A1F11	Beim Lesen lerne ich, bestimmte Typen von Personen oder Ereignissen leichter zu erkennen, d. h. ich kann diese Typen deutlicher ausmachen, nachdem ich ein bestimmtes Beispiel in einem literarischen Text gesehen habe.	In my reading, I learn to recognize more readily certain types of people or events, i.e., I can see these types more clearly after reading about a particular example in a literary text.
A1F12	Wenn ich anfange, einen literarischen Text zu verstehen, dann deshalb, weil ich ihn auf das beziehen kann, was mich im eigenen Leben beschäftigt.	When I begin to understand a literary text, it's because I've been able to relate it to my own concerns about life.
A1F13	In der Literatur werden häufig diejenigen Dinge besonders hervorgehoben, die eine moralische Aussage treffen.	Literature often gives special emphasis to those things that make a moral point.
A1F14	Das Lesen von Literatur löst bei mir manchmal Gefühle aus, die mit einer ausgesprochen beunruhigenden Sicht auf das Leben einhergehen.	Sometimes while reading literature my feelings draw me toward a distinctly unsettling view of life.
A2	**Empathie**	**Empathy**
A2F1	Manchmal habe ich fast das Gefühl, dass ich zu einer Figur „geworden" bin, über die ich in einer Geschichte gelesen habe.	Sometimes I feel like I've almost "become" a character I've read about in fiction.
A2F2	Ich führe manchmal imaginäre Gespräche mit Menschen aus einer Geschichte.	I sometimes have imaginary dialogues with people in fiction.
A2F3	Wenn ich eine Geschichte lese, denke ich oft an mich selbst als eine der Personen in der Geschichte.	When I read fiction I often think about myself as one of the people in the story.
A2F4	Ich frage mich manchmal, ob ich wirklich etwas erlebt habe oder ob ich darüber in einem Buch gelesen habe.	I sometimes wonder whether I have really experienced something or whether I have read about it in a book.

A2F5	Ich versuche aktiv, mich in die Rolle der fiktiven Figuren hineinzuversetzen; fast so, als würde ich mich darauf vorbereiten, in einem Stück mitzuspielen.	I actively try to project myself into the role of fictional characters, almost as if I were preparing to act in a play.
A2F6	Manchmal werden Figuren in Romanen fast zu echten Menschen in meinem Leben.	Sometimes characters in novels almost become like real people in my life.
A2F7	Nachdem ich einen Roman oder eine Geschichte gelesen habe, die mir gefallen hat, mache ich mir weiterhin Gedanken über die Figuren; fast so, als wären sie echte Menschen.	After reading a novel or story that I enjoyed, I continue to wonder about the characters almost as though they were real people.
A3	**Bildliche Deutlichkeit**	**Imagery Vividness**
A3F1	Ich sehe die Orte in Geschichten, die ich lese, oft so deutlich, als würde ich ein Bild betrachten.	I often see the places in stories I read as clearly as if I were looking at a picture.
A3F2	Ich kann mir leicht die Personen und Orte vorstellen, die in einem Roman oder einer Kurzgeschichte beschrieben sind.	I can readily visualize the persons and places described in a novel or short story.
A3F3	Ich denke manchmal, ich könnte eine Karte der Orte zeichnen, über die ich in einer Geschichte gelesen habe.	I sometimes think I could draw a map of the places I have read about in a work of fiction.
A3F4	Manchmal ist eine Szene in einer Geschichte oder einem Gedicht so klar, dass ich ihren Geruch, ihre Berührung, ihre Atmosphäre „spüre".	Sometimes a scene from a story or poem is so clear that I know its smell, its touch, its "feel".
A3F5	In einem Roman höre ich Gespräche oft so, als würde ich einem tatsächlichen Gespräch zuhören.	I often hear dialogue in a novel as though I were listening to an actual conversation.
A3F6	Wenn ich einen literarischen Text lese, wird eine Szene, die nur teilweise beschrieben wird, oft zu einem vollständigen, lebendigen Ort in meinem Kopf.	When I read a literary text, a scene that is only partly described often becomes a whole, vividly present place in my mind.
A3F7	Wenn ich eine Geschichte lese, kann ich manchmal fast spüren, wie es wäre, dort zu sein.	When reading a story, sometimes I can almost feel what it would be like to be there.
A3F8	Normalerweise höre ich den Tonfall in einem Gespräch aus einer Geschichte oder einem Roman.	I usually hear the tone of speech in a dialogue from a story or novel.
A3F9	Wenn ich literarische Texte lese, deuten Geruchsbeschreibungen oft auf Farben hin, Farbbeschreibungen auf Gefühle usw.	Often when I read literary texts, descriptions of smells suggest colors, descriptions of colors suggest feelings, and so on.
A4	**Freizeitflucht**	**Leisure Escape**
A4F1	Manchmal möchte ich es mir mit einem guten Buch gemütlich machen, einfach um mich zu vergnügen.	Sometimes I like to curl up with a good book just to enjoy myself.

A4F2	In meiner Freizeit lese ich am liebsten einen Roman.	When I have spare time my favorite activity is reading a novel.
A4F3	Sehr oft kann ich eine Geschichte nicht weglegen, bevor ich sie zuende gelesen habe.	Very often I cannot put down a story until I have finished reading it.
A4F4	Das Lesen von Literatur ist eine angenehme Art, Zeit zu verbringen, wenn ich nichts anderes zu tun habe.	Reading literature is a pleasurable way to spend time when I have nothing else to do.
A4F5	Das Lesen einer Geschichte ist eine wunderbare Möglichkeit, sich zu entspannen.	Reading a story is a wonderful way to relax.
A4F6	Beim Lesen vergesse ich völlig, wie spät es ist.	While reading I completely forget what time it is.
A4F7	Ich finde, dass das Lesen von Literatur eine große Hilfe ist, um mich von meinen eigenen Problemen abzulenken.	I find that reading literature is a great help in taking my mind off my own problems.
A4F8	Ich mag es, mich so in die Welt eines literarischen Textes zu vertiefen, dass ich meine alltäglichen Sorgen vergesse.	I like to become so absorbed in the world of the literary text that I forget my everyday concerns.
A4F9	Sobald ich ein Werk eines Autors entdeckt habe, den ich mag, versuche ich normalerweise, alle anderen Werke dieses Autors zu lesen.	Once I've discovered one work by an author I like, I usually try to read all the other works by that author.
A4F10	Ich bin oft so in das, was ich lese, hineingezogen, dass ich mir meiner selbst nicht mehr bewusst bin.	I am often so involved in what I am reading that I am no longer aware of myself.
A4F11	Ich wünschte oft, ich hätte mehr Zeit zum Lesen von Literatur.	I often wish I had more time for reading literature.
A5	**Beschäftigung mit dem Autor**	**Concern With Author**
A5F1	Eines meiner Hauptinteressen beim Lesen von Literatur ist es, die Themen und Anliegen eines bestimmten Autors kennenzulernen.	One of my primary interests in reading literature is to learn about the themes and concerns of a given author.
A5F2	Beim Lesen konzentriere ich mich gerne darauf, wodurch sich der Stil des Autors auszeichnet.	In reading I like to focus on what is distinctive about the author's style.
A5F3	Eines meiner Hauptinteressen beim Lesen ist es, die verschiedenen Genres der Literatur kennenzulernen.	One of my primary interests in reading is to learn about the different genres of literature.
A5F4	Ich möchte verstehen, wie sich die Arbeit eines bestimmten Autors auf andere Werke aus der Zeit des Autors bezieht.	I like to see how a particular author's work relates to other literature of the author's period.
A5F5	Beim Lesen versuche ich normalerweise, die unterschiedlichen Themen eines Autors zu identifizieren.	When reading I usually try to identify an author's distinctive themes.

A5F6	Eines meiner Hauptinteressen beim Lesen von Literatur ist es, das Verständnis des Autors für Gesellschaft und Kultur kennenzulernen.	One of my primary interests in reading literature is to appreciate the author's understanding of society and culture.
A5F7	Ich finde Literatur besonders interessant, wenn sie Fakten über das Leben des Autors beleuchtet.	I think literature is especially interesting when it illuminates facts about the author's life.
A5F8	Wenn ich ein literarisches Werk finde, das mir gefällt, versuche ich normalerweise, etwas über den Autor herauszufinden.	When I find a work of literature I like, I usually try to find out something about the author.
A5F9	Die Herausforderung bei Literatur besteht darin, die einzigartige Sicht eines Autors auf das Leben zu verstehen.	The challenge of literature is to comprehend the author's unique view of life.
A5F10	Ich bin oft fasziniert von der literarischen Technik eines Autors.	I am often intrigued by an author's literary technique.
A6	**Handlungsorientiertes Lesen**	**Story-Driven Reading**
A6F1	Ich mag es, wenn sich in der Handlung einer Geschichte Spannung aufbaut.	I like to see tension building up in the plot of a story.
A6F2	Die Art von Literatur, die mir am besten gefällt, erzählt eine interessante Geschichte.	The type of literature I like best tells an interesting story.
A6F3	Ich denke, dass der wichtigste Teil einer Geschichte oder eines Dramas die Handlung ist.	I think the most important part of fiction or drama is plot.
A6F4	Wenn ich einen Roman lese, möchte ich vor allem wissen, wie die Geschichte ausgeht.	When reading a novel, what I most want to know is how the story turns out.
A6F5	Mir gefällt es am besten, wenn eine Geschichte ein unerwartetes Ende hat.	I like it best when a story has an unexpected ending.
A6F6	Ich lese lieber Geschichten, in denen es viel Action gibt.	I prefer to read fiction in which there is plenty of action.
A6F7	Beim Lesen eines Romans ist mein Hauptinteresse, zu sehen, was mit den Figuren passiert.	When reading a novel my main interest is seeing what happens to the characters.
A6F8	Ich finde es schwierig, einen Roman zu lesen, in dem nicht viel zu passieren scheint.	I find it difficult to read a novel in which nothing much seems to happen.
A7	**Ablehnung literarischer Werte**	**Rejection of Literary Values**
A7F1	Ich denke, dass die Leute weniger Zeit damit verbringen sollten, über Literatur zu sprechen oder zu schreiben.	I think people should spend less time talking or writing about literature.
A7F2	Selbst wenn der Unterricht von Literatur gut umgesetzt wäre, sollten die Schulen meiner Meinung nach nicht so viel Zeit darauf verwenden.	Even if literature were well taught, I think high schools should not devote so much time to it.
A7F3	Für mich wird ein literarisches Werk zerstört, wenn man versucht, es zu analysieren.	For me a work of literature is destroyed by trying to analyze it.

A7F4	Eines der Dinge, die ich am Unterricht von Literatur am wenigsten mag, ist der Lehrer, der einem sagt, was ein literarischer Text bedeutet.	One of the things I dislike most about being a student of literature is the teacher who tells you what a literary text means.
A7F5	Das Lesen von literarischen Texten aus vergangenen Jahrhunderten sollte Literaturwissenschaftlern und Historikern überlassen bleiben.	Reading literary texts from past centuries should be left to literary scholars and historians.
A7F6	Ich glaube nicht, dass Literatur gesellschaftlich relevant ist.	I don't believe that literature is socially relevant.
A7F7	Ich mochte Deutsch in der Schule nicht, weil ich die meisten Texte, die ich lesen sollte, nicht selbst ausgewählt hätte.	I disliked English in high school because most of the texts I was asked to read I would not have chosen myself.
A7F8	Literarische Werke scheinen die Themen des Lebens oft komplizierter zu machen, als sie tatsächlich sind.	Works of literature often seem to make the issues of life more complicated than they actually are.
A7F9	Wenn ich Zeit mit Lesen verbringen möchte, entscheide ich mich nicht für „literarische" Texte.	If I want to spend time reading, I don't choose "literary" texts.
Part B	***Original used in this work***	***Translation for English readers***
B1	**Präferenzen für manifeste Erzähler**	**Preferences for Manifest Narrators**
B1F1	Ich lese gerne Romane, die einen Ich-Erzähler haben.	I like to read novels that have a first-person narrator.
B1F2	Ich lese gerne Romane, in denen der Erzähler häufig das Geschehen kommentiert.	I like to read novels in which the narrator often comments on the action.
B1F3	Ich lese gerne Romane, in denen der Erzähler oft in den Vordergrund tritt und die Geschichte dahinter zurücktritt.	I like to read novels in which the narrator often comes to the fore and the story takes a back seat.
B2	**Autor als Auswahlkriterium für Literatur**	**Author as Criterion for Selecting Literature**
B2F1	Ich lese gerne autobiographische Romane.	I like to read autobiographic novels.
B2F2	Ich lese gerne Literatur, die ein bekannter Autor geschrieben hat.	I like to read literature that is written by a famous author.
B2F3	Ich wähle meine Lektüre vor allem nach dem Autor aus.	I select my reading most of all by the author.
B3	**Text als Kommunikat des Autors**	**Text as Message of the Author**
B3F1	Mit einem Roman möchte der Autor seinen Lesern etwas vermitteln.	The author wants to convey something to the readers with a novel.
B3F2	Eine Geschichte spiegelt die Weltsicht des Autors wider.	A story reflects the author's worldview.
B3F3	Wenn ich ein literarisches Werk lese, bekomme ich eine Vorstellung von seinem Autor.	When I read a literary work, I get an idea of its author.
B4	**Pan-Narrator-Theory**	**Pan-Narrator Theory**

B4F1	Der Erzähler in einem Roman ist niemals der Verfasser des Romans.	The narrator in a novel is never its writer.
B4F2	Jeder Roman hat einen Erzähler, den man nicht mit dem Autor gleichsetzen sollte.	Every novel has a narrator, which should not be put on a level with the author.
B4F3	Der Erzähler ist im Roman vorhanden, auch wenn er nicht extra erwähnt wird.	There is a narrator in a novel even if not being mentioned specifically.
B5	**Implizite Autorinstanz**	**Implicit Author**
B5F1	Wenn ich einen Roman lese, entsteht in meinem Kopf ein Bild des Autors, das nicht dem realen Autor entsprechen muss.	When reading a novel, an image of the author emerges in my mind, which does not necessarily correspond to the real author.
B5F2	Wenn beim Lesen eines Romans ein Bild des Autors in meinem Kopf entsteht, interessiert mich nicht, ob es dem realen Autor entspricht.	When an image of the author emerges in my mind while reading, I am not interested in whether it corresponds to the real author.
B5F3	Ich mache mir beim Lesen eines literarischen Textes klar, dass meine Vorstellung vom Autor dem realen Autor nicht unbedingt ähnlich ist.	When reading a literary text, I bring to my mind that my idea of the author does not necessarily resemble the real author.
B6	**Enge Beziehung zwischen Autor und Figur**	**Close Relation between Author and Character**
B6F1	Ich stelle mir manchmal vor, dass der Autor sich selbst in eine Figur geschrieben hat.	Sometimes I imagine that the author has written himself into a character.
B6F2	Ich stelle mir manchmal vor, dass eine Figur die Überzeugungen des Autors wiedergibt.	Sometimes I imagine that a character reflects the author’s beliefs.
B6F3	Bei manchen Figuren stelle ich mir vor, dass der Autor ähnliche Erfahrungen gemacht hat.	With some characters, I imagine that the author has had similar experiences.
B7	**Enge Beziehung zwischen Autor und Erzähler**	**Close Relation between Author and Narrator**
B7F1	Ich stelle mir manchmal vor, dass der Autor sich selbst in den Erzähler geschrieben hat.	Sometimes I imagine that the author has written himself into the narrator.
B7F2	Ich stelle mir manchmal vor, dass der Erzähler die Überzeugungen des Autors wiedergibt.	Sometimes I imagine that the narrator reflects the author’s beliefs.
B7F3	In Bezug auf den Erzähler stelle ich mir manchmal vor, dass der Autor ähnliche Erfahrungen gemacht hat.	Regarding the narrator, I sometimes imagine that the author has had similar experiences.
[no group]	**Füllfragen**	**Filler Questions**
B8F1	Ich schreibe Geschichten lieber mit Füller als mit Bleistift.	I prefer to write stories with pen rather than pencil.
B8F2	Ich kaufe Bücher lieber neu als gebraucht.	I prefer to buy new books rather than used ones.
B8F3	Ich lese lieber elektronisch als auf Papier.	I prefer to read electronically rather than on paper.
B8F4	Ich lese lieber Hardcover als Softcover.	I prefer to read hardcover rather than softcover.

B8F5	Ich lese gerne das Buch zum Film.	I like to read the book on the film.

Table S2. Questions as given to the annotators (left) and the corresponding English version (right).

REFERENCES

Miall, D. S. and Kuiken, D. (1995). Aspects of literary response: A new questionnaire. *Research in the Teaching of English* , 37–58
